# Zeolite-supported ultra-small nickel as catalyst for selective oxidation of methane to syngas

**DOI:** 10.1038/s42004-020-00375-0

**Published:** 2020-09-16

**Authors:** Shuhei Yasuda, Ryota Osuga, Yusuke Kunitake, Kazuya Kato, Atsushi Fukuoka, Hirokazu Kobayashi, Min Gao, Jun-ya Hasegawa, Ryo Manabe, Hisashi Shima, Susumu Tsutsuminai, Toshiyuki Yokoi

**Affiliations:** 1 Institute of Innovative Research, Tokyo Institute of Technology, 4259 Nagatsuta, Midori-ku Yokohama, 226-8503 Japan; 2 Institute for Catalysis, Hokkaido University, Kita 21 Nishi 10, Kita-ku Sapporo, 001-0021 Japan; 3 Mitsubishi Chemical Corporation, 1000 Kamoshida-cho, Aoba-ku Yokohama, 227-8502 Japan

**Keywords:** Catalysis, Materials chemistry, Materials for energy and catalysis, Nanoscale materials

## Abstract

The development of simple catalysts with high performance in the selective oxidation of methane to syngas at low temperature has attracted much attention. Here we report a nickel-based solid catalyst for the oxidation of methane, synthesised by a facile impregnation method. Highly dispersed ultra-small NiO particles of 1.6 nm in size are successfully formed on the MOR-type zeolite. The zeolite–supported nickel catalyst gives continuously 97–98% methane conversion, 91–92% of CO yield with a H_2_/CO ratio of 2.0, and high durability without serious carbon deposition onto the catalyst at 973 K. DFT calculations demonstrate the effect of NiO particle size on the C-H dissociation process of CH_4_. A decrease in the NiO particle size enhances the production of oxygen originating from the NiO nanoparticles, which contributes to the oxidation of methane under a reductive environment, effectively producing syngas.

## Abstract (web summary)

Catalytic oxidation of methane to syngas is an important process, but efficient and stable catalyts remain in demand. Here dispersion of nickel on zeolites yields nickel oxide nanoparticles as small as 1.6 nm which act as selective and efficient catalysts for conversion of methane to syngas.

## Introduction

Methane, the simplest alkane, is the principal component of natural gas and shale gas, which is a highly abundant and inexpensive carbon source of fuel and chemicals. Therefore, a development of the catalytic process that can directly convert methane into valuable chemicals leads to the effective use of methane as carbon source, and intensive efforts have been devoted to develop this catalytic process^1–7^. However, a technological breakthrough has been remained. The approach of conversion of methane to syngas is mostly based on steam reforming^8,9^ and dry reforming^10,11^. These steam and dry reforming reactions are strongly endothermic; higher temperature and/or higher pressure of CH_4_ are required. On the other hand, neither higher temperature nor higher pressure is required for oxidation of methane to CO and H_2_^12,13^.

Recently, various catalysts with precious and/or nonprecious metals for selective oxidation of CH_4_ to CO and H_2_ productions have been proposed^12–21^. In previous work, Kobayashi et al. have reported a high performance using Rh/zeolite catalyst^18^; Rh sub-nano clusters are formed on the MOR-type zeolite and this Rh/MOR catalyst gave 84% CH_4_ conversion and 91% selectivity for CO with H_2_/CO ratio of 2.0 at 873 K^18^. In addition, when cobalt was added to the catalyst, the catalytic performance was improved^19^. Highly dispersed and mono-atomically controlled Rh species in Rh-Co/zeolite enhanced the reduction of Co species followed by the redox cycle of the catalyst, leading to the improvement.

Although various supported metal catalysts including Rh, Ni, and Rh-Ni, have been intensively developed for methane conversion, the catalytic application of supported Ni catalyst in the selective oxidation of methane to syngas has not been reported to date. Hence, the control of the Ni particle size and its effect on the performance have not been investigated to date. Furthermore, the mechanism of selective oxidation of methane to syngas over supported metal catalysts has not been completely clarified. The selective oxidation of methane is governed by the reaction equilibrium limitation involving CH_4_, CO, CO_2_, H_2_, and H_2_O. In order to achieve the production of syngas with a high yield at low temperature, the development of a novel catalyst that can overcome the equilibrium limitation has strongly been desired.

Here, a preparation method that can achieve the controls of the particle size of Ni species and its dispersion on zeolite has been developed based on the incipient wetness technique. Thus prepared zeolite-supported Ni catalyst is a highly active, selective and durable for oxidation of methane to syngas. In addition, DFT calculations demonstrate the size effect on the catalytic activity of NiO nanoparticles. Furthermore, the reaction mechanism involving lattice oxygen in ultra-small sized NiO species has been proposed.

## Results and discussion

### Size effect on the catalytic activity of NiO nanoparticles

**Fig. 1:**
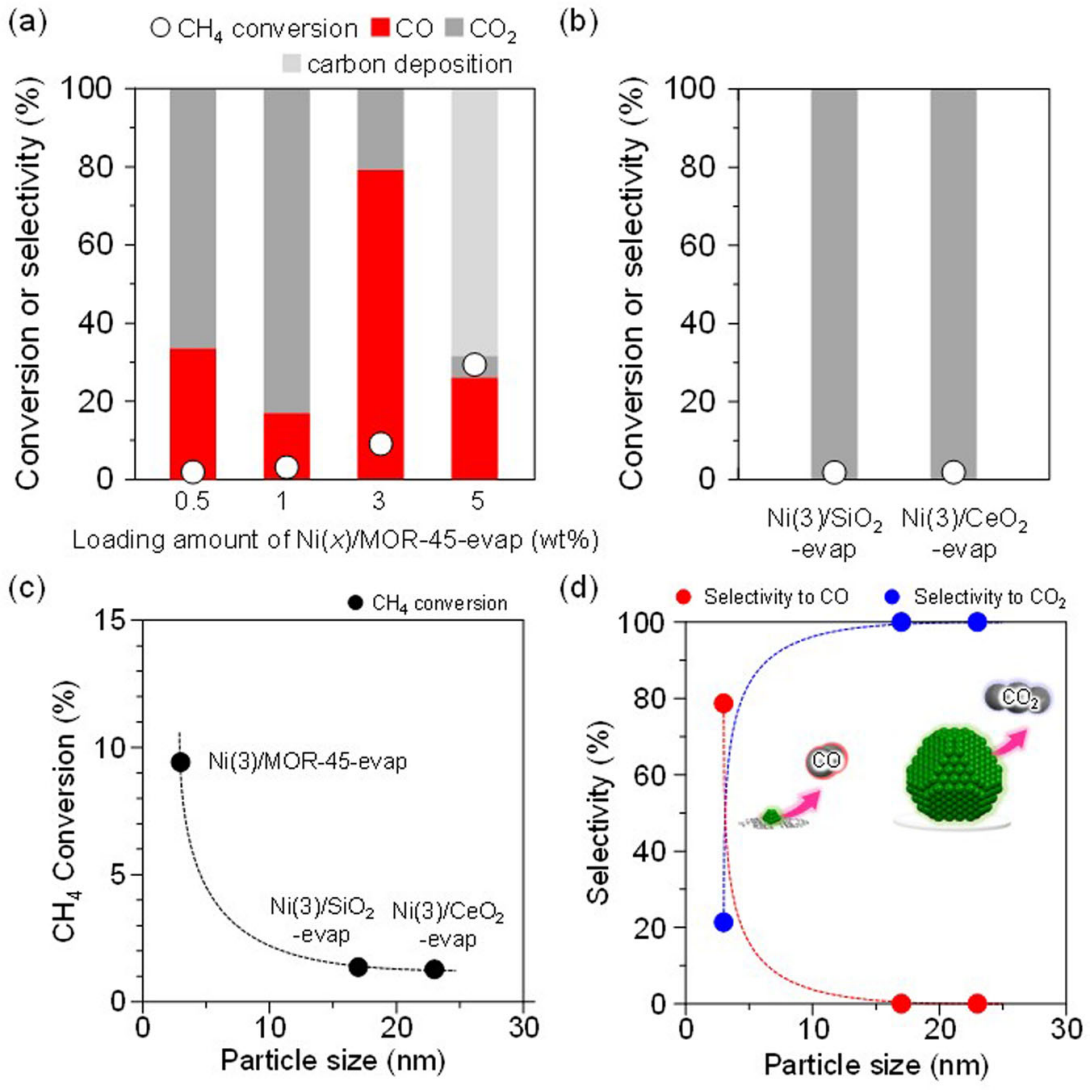
Oxidation of methane over supported Ni catalysts. **a** Ni(*x*)/MOR-45-evap and **b** metal oxides–supported Ni catalysts, Ni(3)/SiO_2_ and Ni(3)/CeO_2_. (○) Conversion of methane, (red and gray) bars represent yield of CO and CO_2_, respectively. Reaction conditions: CH_4_:O_2_:N_2_ = 0.50:0.04:0.46 (atm); total pressure, 0.1 MPa; temperature, 873 K; and SV = 6.0 × 10^4^ mL h^−1^ g_-cat_^−1^. The catalytic performance plotted against Ni nanoparticle size for methane oxidation ((**c**) and **d**)). A second–order polynomial fit (broken–line) is drawn through the points as an eye guide. These catalytic results were summarized in Supplementary Table 1.

It has been reported that the large size of Ni particle promotes decomposition of CH_4_, namely coke formation, in the absence of O_2_^22^. Very recently, the importance of Ni particle size on dry reforming and steam reforming of methane has been reported^23^. First, the effect of the loading amount of Ni was investigated; Ni(*x*)/MOR-45 was prepared with *x* varied ranging from 0.5 to 5 wt% (Supplementary Fig. 1). The results are summarized in Supplementary Table 1. The irregular change in the selectivity to CO over Ni(0.5)/MOR-45-evap and Ni(1)/MOR-45-evap would be due to the low conversion of CH_4_. Catalytic properties of the prepared samples were checked under the methane-rich reaction conditions, where coke resistance ability is easily evaluated. Ni(5)/MOR-45-evap showed a much lower catalytic performance than the catalyst prepared by the same method but having particle size of 3 nm [Ni(3)/MOR-45-evap] (Fig. 1a). Moreover, Ni(5)/MOR-45-evap caused serious carbon deposition (21% yield), indicating decomposition of CH_4_ on large Ni species. The effect of particle size including that within the same support (MOR-45), we prepared Ni(5)/MOR-45-evap, Ni(3)/SiO_2_-evap and Ni(3)/CeO_2_-evap, having Ni species 15–25 nm in size, by a typical impregnation method based on the evaporation technique (Supplementary Fig. 2). For Ni(3)/SiO_2_-evap and Ni(3)/CeO_2_-evap, the conversions of CH_4_ were significantly low (ca. less than 1%), and only slight amounts of CO_2_ were produced (Fig. 1b). Due to the very low activity, even coking did not occur. These results strongly give an insight about size-dependent activity relationships for the selective oxidation of CH_4_ to CO (Fig. 1c, d), though the influence of support might not be negligible.

DFT calculations were conducted to clarify the size effect on the catalytic activity of NiO nanoparticles. The C-H dissociation process of CH_4_ was investigated because it has commonly been accepted as the first and a difficult step for oxidation of methane. The NiO surface and NiO cluster were chosen as the representative models for large- and small-sized NiO particles. It is known that the low Miller index surfaces, such as (100), (110), and (111) are stable and have been extensively considered in catalytic reactions. Here, we considered these three kinds of surfaces and compared their stabilities. The NiO(100) surface yields a nonpolar plane with the lowest surface energies, which means the NiO(100) surface would be the main facet for large size particles^24^. Therefore, we chose NiO(100) surface as the computational model for the large sized particles.

As shown in Supplementary Fig. 3, CH_4_ can bind weakly on the NiO(100) and NiO cluster with adsorption energies of −0.17 eV and −0.13 eV, respectively. While the C-H dissociation is endothermic on NiO(100) with the reaction energies of 1.7 eV, the reaction on NiO cluster is exothermic with the reaction energy of −0.68 eV. After the C-H dissociation, CH_3_ and H species formed the bonds with terrace Ni and O atoms of NiO(100) surface, but with the ones at edge/corner sites of NiO cluster. Compared to the terrace atoms, the edge/corner ones are low-coordinated and supposed to be more active. Thus, these calculations suggest that the ratio of edge/corner atoms over terrace atoms increases by decreasing the size of NiO nanoparticles, leading to the improvement of the catalytic activity.

### Synthesis and reactivity of supported ultra-small Ni catalyst

**Fig. 2:**
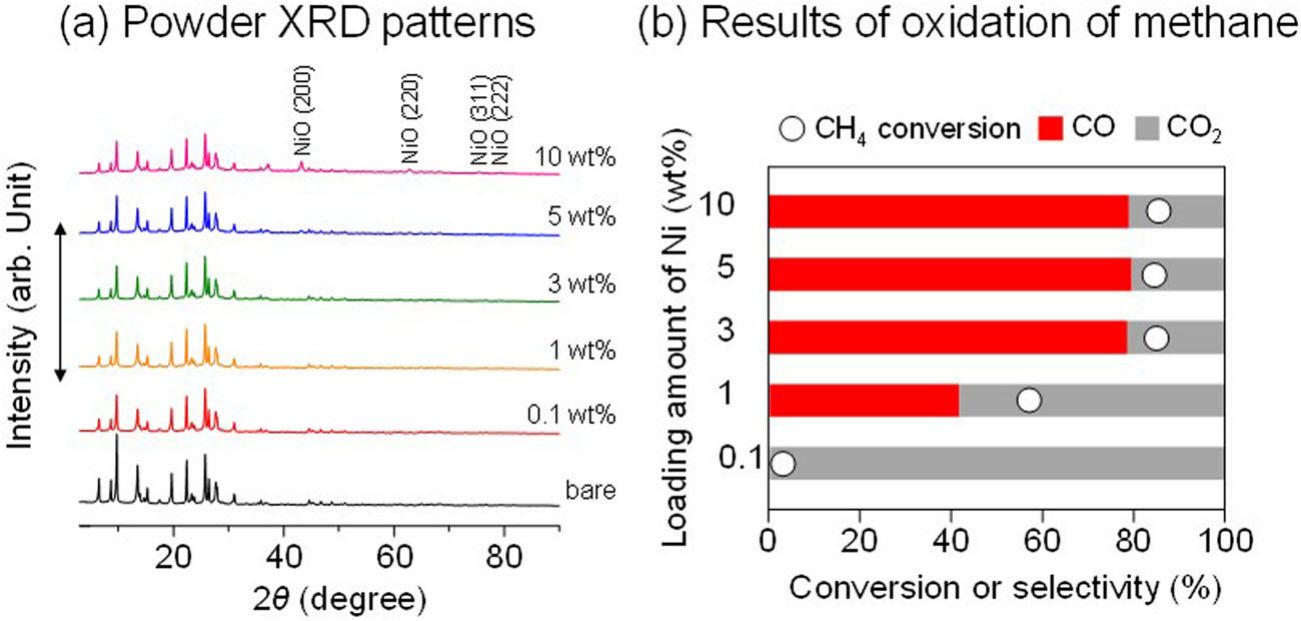
Effect of the loading amounts of Ni on MOR-45. **a** XRD patterns of Ni(*x*)/MOR-45 and **b** Oxidation of methane over Ni(*x*)/MOR-45: (○) Conversion of methane, (red and gray) bars represent the yields of CO and CO_2_, respectively. Reaction conditions: CH_4_:O_2_:Ar = 0.06:0.03:0.91 (atm); total pressure, 0.1 MPa; temperature, 873 K; and SV = 3.0 × 10^4^ mL h^−1^ g_-cat_^−1^. Catalytic data were taken after at least 3 h from the beginning of the reaction.

**Fig. 3:**
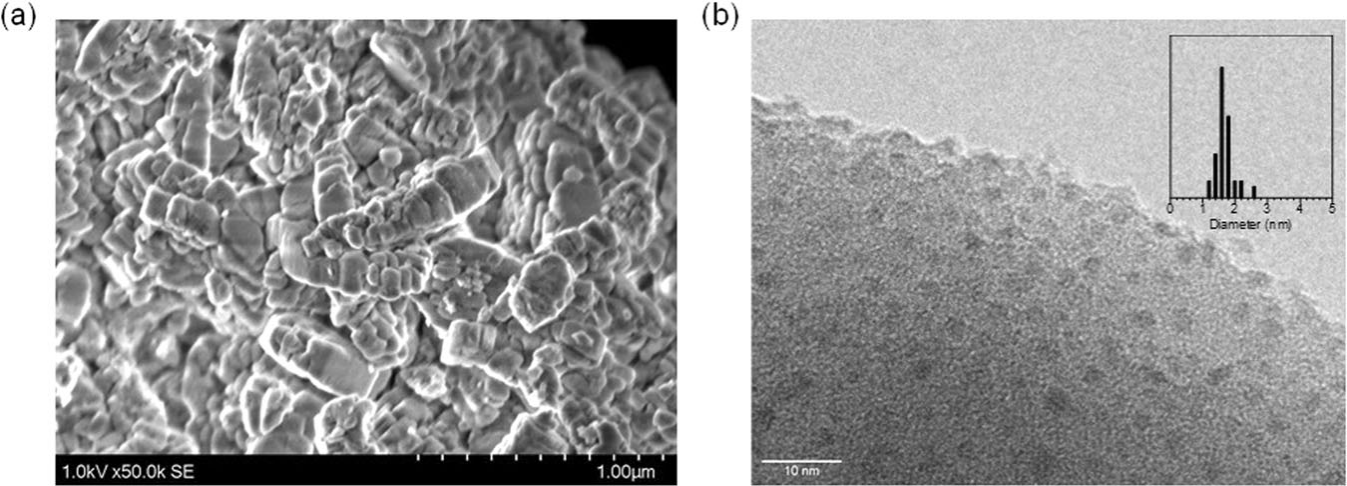
Images of Ni(5)/MOR-45. **a** SEM and **b** TEM images.

The effect of the loading amount of Ni was investigated; Ni(*x*)/MOR-45 was prepared with *x* varied ranging from 0.1 to 10 wt%. All the samples of Ni(*x*)/MOR-45 had a highly crystalline MOR-type structure (Fig. 2a). At *x* = 0.1–5, the diffraction peaks attributed to NiO species were hardly observed, suggesting that the Ni species were highly dispersed on the surface of MOR-45. However, at *x* = 10, the diffraction peaks attributed to the NiO species such as 2*θ* = 43° (200), 63° (220), 75° (311) and 79° (222) planes were slightly observed^25^. Ni(5)/MOR-45 was reprehensively characterized by SEM and TEM observations. The SEM image revealed that bulky Ni particles were not observed on the surface of the zeolite (Fig. 3a). The TEM image was employed to determine the particle size of the Ni species, indicating that the particles with a mean size of 1.6 nm were highly distributed in the MOR zeolite(Fig. 3b and Supplementary Fig. 4). In combination with the EDX analyses (Supplementary Fig. 5), the Ni species were highly dispersed on the zeolite. As a control, Ni(*x*)/MOR-45 were prepared by a typical impregnation method based on the evaporation technique, and thus prepared samples were designated as “Ni(*x*)/MOR-45-evap” (Supplementary Fig. 1). At *x* = 3, Ni particles 3 nm in size were formed. However, unlike the incipient wetness technique, the Ni loading of 5 wt% resulted in the formation of the Ni particles 17 nm in size (Supplementary Table 1). These results clearly demonstrated that the incipient wetness technique is an effective method for the introduction of nano-sized and highly dispersed Ni species onto zeolite even at high loading.

The catalytic performance of thus prepared catalysts in oxidation methane with a CH_4_/O_2_ ratio of 2, which is based on the stoichiometric ratio for syngas production (CH_4_ + 1/2O_2_ → CO + 2H_2_), were evaluated (Fig. 2b). The loading amount of Ni had a great impact on the catalytic performance. The conversion of CH_4_ was increased along with *x*, and it reached about 80% after *x* = 3. The increase in *x* also led to the decrease in the selectivity to CO_2_. Ni(5)/MOR-45 exhibited the highest performance; it gave 85% of the conversion of CH_4_ and 79.6% of the selectivity to CO with the H_2_/CO ratio, 2.3. When Ni loading was further increased to 10 wt%, the conversion of CH_4_ was slightly increased to 82%, but the selectivity to CO decreased to 78.9 with the H_2_/CO ratio of 2.2 compared to Ni(5)/MOR-45.

The influences of Al content in the zeolite on the state of Ni species loaded and the catalytic performance were investigated. The catalysts with the Si/Al ratio varied were prepared with the Ni loading amount kept (*x* = 5); the catalyst was designated as “Ni(5)/MOR-*y*,” where *y* was the Si/Al ratio between 7.5 and 120. There was little change in the X-ray diffraction (XRD) patterns of Ni(5)/MOR-*y* (Supplementary Fig. 6). The catalytic performance was not significantly influenced by the Al content in the zeolite; the conversion was about 82–84%, and the selectivity to CO was 78–80% irrespective of *y* (Supplementary Fig. 7). These results imply that the Al content is not a definitive factor for the formation of the active species. We also tested other zeolites including Ni(5)/MFI-45 and Ni(5)/FER-10 as a representative zeolite for methane oxidation (Supplementary Fig. 8*)*. These catalysts gave almost the same catalytic performance as Ni(5)/MOR.

Here we showed you the advantages of the utilization of zeolite and the impact of Ni size on the catalytic performance. As a control, 5 wt% of Ni species were similarly loaded onto amorphous silica-alumina and silica; thus obtained catalysts were Ni(5)/SiO_2_-Al_2_O_3_ and Ni(5)/SiO_2_, respectively. In particular, the diffraction peaks attributed to NiO species Ni(5)/SiO_2_ were clearly observed in the XRD pattern of Ni(5)/SiO_2_ (Supplementary Fig. 9). Note that their catalytic performance showed lower than that of zeolite as the support (Supplementary Fig. 10). In the case of Ni(5)/SiO_2_-Al_2_O_3_, Ni-aluminate species such as NiAl_2_O_4_ were formed on the surface and it is considered to be inert to the reaction. There were no significant changes in the XRD patterns and ^27^Al MAS NMR spectra of before and after the reaction at 873 K (Supplementary Fig. 11a, b), indicating that all the Al species remained intact in the MOR framework even after the reaction, and thus, the high stability of the Al species suppress the formation of the Ni-aluminate species. Furthermore, as a control, the utilizations of inert “silica” and “ceria” with redox property as support showed a low catalytic activity compared to Ni(5)/MOR-45. Thus, we have concluded that acidic and base properties of support are not a definitive factor for this reaction, but the NiO particle size. These results imply that one of advantages of the utilization of zeolite is the control of the Ni species loaded, i.e., the formation of highly dispersed nano-sized Ni species, leading to a high catalytic performance. Thus our findings strongly indicate that the utilization of zeolite as support enables us to control of the size of Ni species and their dispersion that are critical factors for the partial oxidation of CH_4_ to syngas.

**Fig. 4:**
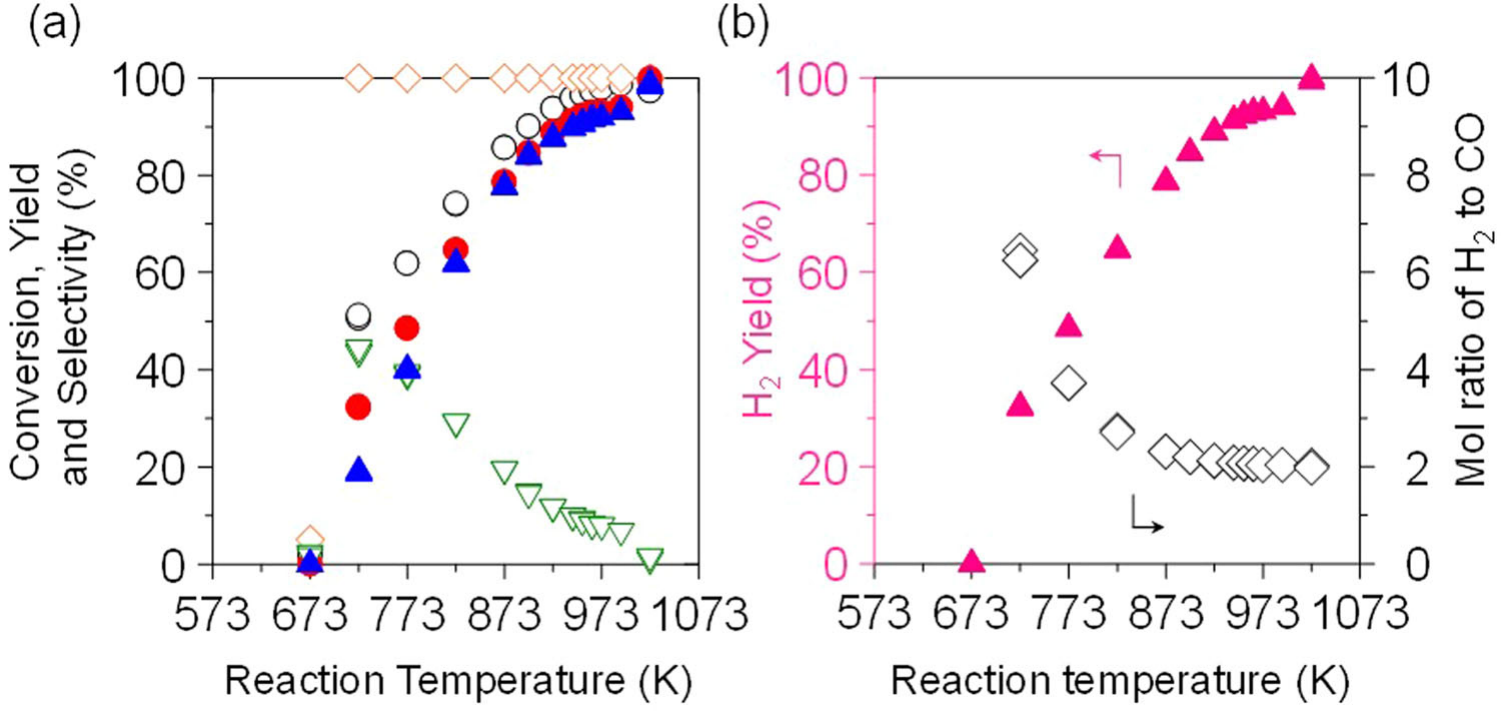
Effect of reaction temperature on the oxidation of methane to syngas over Ni(5)/MOR-45. **a** Conversions of (○) methane and (◊) oxygen, yields of (●) CO, (∇) CO_2_, and (▲) selectivity for CO. **b** (▲) Yield of H_2_ and (◊) mol ratio of H_2_ to CO. Reaction conditions: CH_4_:O_2_:Ar = 0.06: 0.03: 0.91 (atm); total pressure, 0.1 MPa; temperature, 673–1023 K; and SV = 3.0 × 10^4^ mL h^−1^ g_-cat_^−1^.

The temperature dependence of the reaction over Ni(5)/MOR-45 between 673 and 1023 K was investigated (Fig. 4). The proportion of CH_4_, CO_2_, CO, and H_2_ were considered based on thermodynamics by comparing with equilibrium composition. At 673 K, the conversions of CH_4_ and O_2_ were 2 and 5%, respectively, and the production of CO was not observed; the main product was CO_2_ (selectivity was >99%), suggesting that only complete oxidation of methane to CO_2_ occurred. When the temperature raised to 723 K, the conversions of CH_4_ and O_2_ were dramatically increased to about 50 and 100%, respectively, and the yield of CO was 30%. After 723 K, the conversion of CH_4_ and yield of CO were gradually increased but the yield of CO_2_ was decreased along with the reaction temperature, and the proportion of CH_4_, CO_2_, CO, and H_2_ became the equilibrium composition. When the reaction temperature was raised to 1023 K, 99% of the selectivity to CO with 2.0 of H_2_/CO ratio was achieved (CO yield 98–99% and CH_4_ conversion almost 100%), meaning that the oxidation of CH_4_ stoichiometrically proceeded and the converted CH_4_ was transformed to CO and H_2_ (CH_4_ + 1/2O_2_ → CO + 2H_2_). In the Rh/MOR catalyst, so-called “volcano-type behavior” was observed for the temperature dependence; the maximum activity was achieved at 873 K^18^. Thus, our catalyst is superior in the production of syngas from CH_4_ to the catalysts reported.

**Fig. 5:**
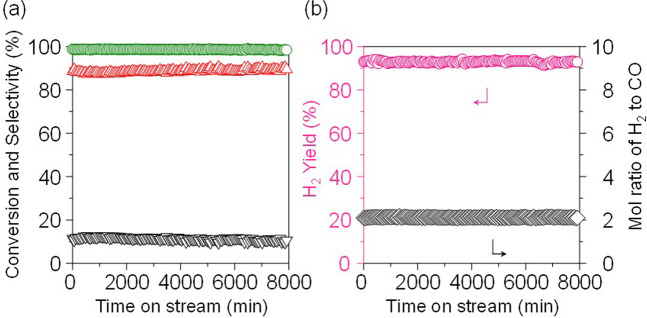
Time courses for the oxidation of methane over Ni(5)/MOR-45. **a** (○) Conversion of methane and selectivities for (△) CO and (∇) CO_2_. **b** (○) Yield of H_2_ and (◊) mol ratio of H_2_ to CO. Reaction conditions: CH_4_:O_2_:Ar = 0.06:0.03:0.91 (atm); total pressure, 0.1 MPa; temperature, 973 K; and SV = 3.0 × 10^4^ mL h^−1^ g_-cat_^−1^. 97–98% methane conversion, 91–92% of CO yield with a H_2_/CO ratio of 2.0 without serious carbon deposition onto the catalyst.

It is worth to note that the stability of the Ni(5)/MOR-45 catalyst was unprecedentedly high. It exhibited stable activity and selectivity for at least 1260 min at 873 K. When the reaction temperature was 973 K, the catalyst gave the highest conversion and selectivity, and the stable activity and selectivity to CO was kept for 8000 min (Fig. 5). The catalyst after the reaction was similarly characterized; the XRD pattern indicated that the crystallinity of the MOR zeolite was slightly decreased, and the SEM image revealed that the particle morphology was slightly destroyed but bulky Ni particles were not observed on the surface of the zeolite. Considering that the Ni(5)/MOR-45 catalyst exhibited a stable performance for a long time, we have considered that a slight decrease in the crystallinity did not affect the catalytic performance (Supplementary Figs. 12 and 13).

### Mechanistic study on oxidation of methane over Ni

**Fig. 6:**
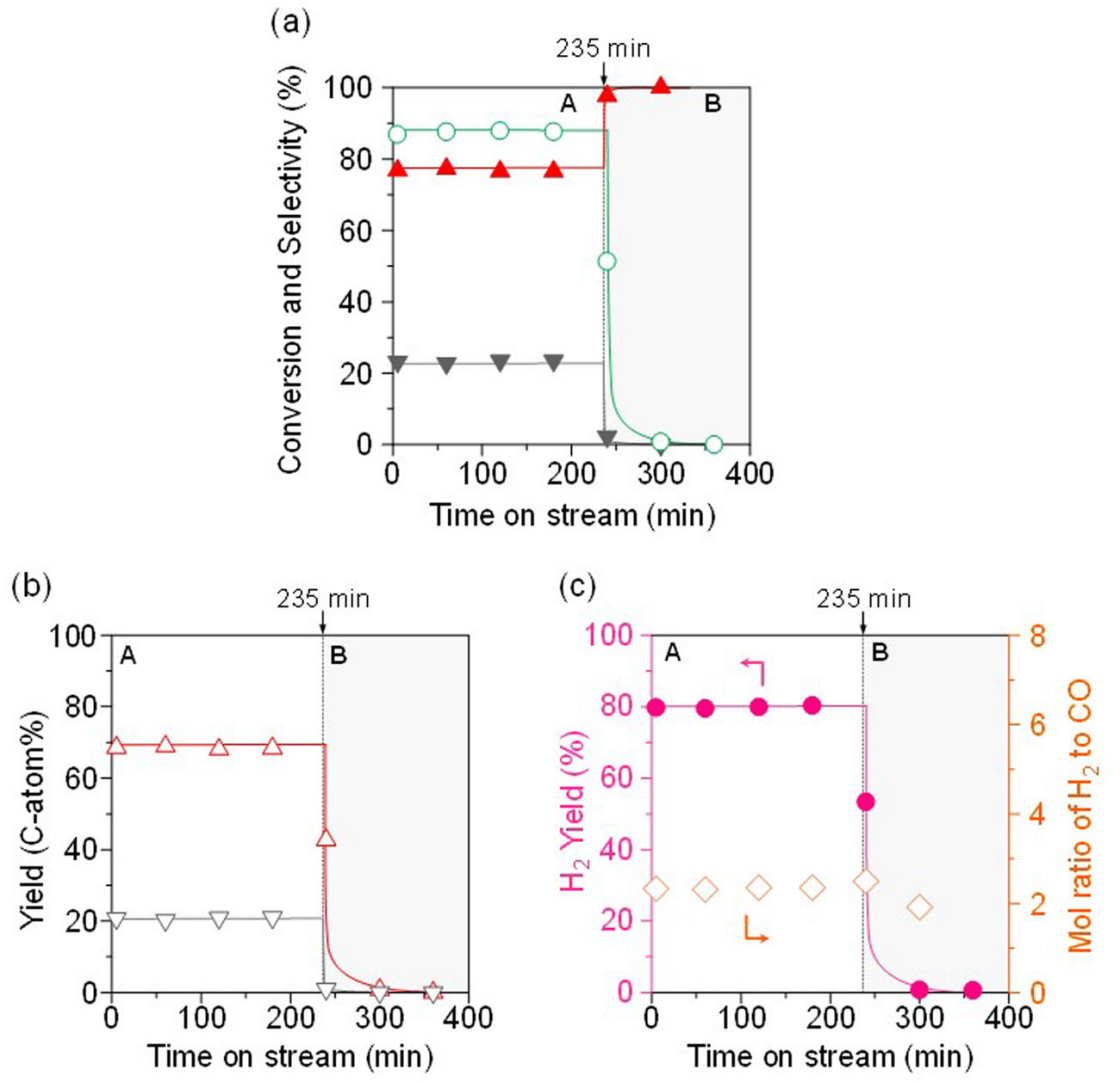
Transient response for the oxidation of methane over Ni(5)/MOR-45 by changing the feed gas. The feed gas was changed from **A**, in the presence of O_2_ ($$P_{O_2} $$ = 0.03 atm) to **B**, in the absence of O_2_ ($$P_{O_2} $$ = 0 atm) at 235 min. **a** (○) Conversion of methane and selectivities for (▲) CO and (▼) CO_2_. **b** Yields of (△) CO and (∇) CO_2_. **c** (●) Yield of H_2_ and (◊) mol ratio of H_2_ to CO. Reaction conditions: CH_4_:O_2_:Ar = (**A**) 0.06:0.03:balance (atm), (**B**) 0.06:0:balance (atm); total pressure, 0.1 MPa; temperature, 873 K; and SV = 3.0 × 10^4^ mL h^−1^ g_-cat_^−1^.

The origin of oxygen introduced into the product, lattice oxygen atom or the oxygen adsorbed on surface, should be clarified to estimate the reaction mechanism. Based on computational chemistry, Nørskov et al. reported that lattice oxygen atom of Ni species plays a dominant role in redox reaction system^26^. It has been considered that, in Rh-Co/MOR, the mono-atomically controlled Rh species with a high dispersion would enhance the reduction of Co species, accelerating the redox-cycle followed by the improvement of the catalytic performance. The oxygen species that can selectively oxidize CH_4_ to CO over Ni(5)/MOR-45 was investigated. We have adopted transient response experiments to investigate the influence of O_2_ in the reaction feed gas stream (Fig. 6). In the initial 235 min, the reaction over Ni(5)/MOR-45 was conducted in the feed with *P*_O2_ = 0.03 atm in steady-state, and then the reaction was switched to that with *P*_O2_ = 0 atm. The reaction was continued even after the stop of O_2_ supply; the productions of CO and H_2_ were observed. Finally, the reaction completely stopped 120 min after the O_2_ supply was turned off. The fact implies that one of the reaction pathways is based on “Mars-van Krevelen mechanism”, in which lattice oxygen of the NiO in Ni/MOR can oxidize methane. To support this mechanism, the catalyst after the transient response reaction was characterized by XRD and X-ray photoelectron (XPS) measurements in terms of the reduction degree of the Ni species. Supplementary Fig. 14 demonstrated that Ni^0^ species were clearly observed in the powder XRD patterns, while NiO species were less observed. It should be noted that the spectra due to Ni 2p_1/2_ and Ni 2p_3/2_ were shifted toward low binding energies (Supplementary Fig. 15). These changes support that the NiO was reduced to Ni when CH_4_ was oxidized to CO. Furthermore, there was a difference in transient response between the CO and CO_2_ yields after the stop of O_2_ supply; the production of CO_2_ was drastically decreased, while that of CO was gradually decreased. Note that, at this transient response, the selectivity to CO and H_2_/CO ratio were continuously near 100% and 2.0, respectively even after stop of O_2_ supply. The CO_2_ produced under the feed with O_2_ might be produced by oxidation by the surface mobile oxygen species^25^. Based on H_2_-TPR results (Supplementary Fig. 16), the surface oxygen species with a high mobility were found to be an oxidant in this methane oxidation, supporting the importance of the reduction conditions during the reaction.

In addition to Ni(5)/MOR-45 (Fig. 6), the transient response experiment for Ni(5)/SiO_2_, which has NiO nanoparticles 15 nm in size, was carried out to clarify the effect of the NiO particles size (Supplementary Fig. 17). The result indicated that the reaction was continued even after the stop of O_2_ supply; the productions of CO and H_2_ were observed. However, it should be noted that the H_2_/CO ratio was increased from 2 to more than 4, and that CO_2_ was also slightly produced. Thus observed differences between Ni(5)/MOR-45 and Ni(5)/SiO_2_ suggest that the NiO particle size strongly affected the catalytic properties and that the decrease in the size enhanced the selective production of syngas from oxidation of methane.

In order to clarify the redox ability depending on the NiO particle size, we carried out the reaction of CH_4_ without O_2_ flow over Ni(5)/MOR-45 and Ni(5)/SiO_2_ (Supplementary Fig. 18). There was no marked difference in the amount of consumed lattice oxygen originated from the NiO nanoparticles between Ni(5)/MOR-45 and Ni(5)/SiO_2_. Note that Ni(5)/MOR-45 only produced CO, while Ni(5)/SiO_2_ produced CO_2_ as well as CO, implying that CO formed is successively oxidized to CO_2_, and that the direct oxidation of CH_4_ to CO_2_ proceeded on the large-sized NiO species. We have considered that the successive oxidation of CO to CO_2_ could be retarded by the reduction of the particle size of NiO species.

We estimated the amount of lattice oxygen in Ni(5)/MOR-45 on the assumption that all the Ni species are present as “NiO”; it was calculated to be 0.76 mmol/g. Besides, the consumption of H_2_ in H_2_-TPR measurement was found to be 0.73 mmol/g, being almost consistent with the amount of lattice oxygen originated from the NiO species. We carried out the reaction of CH_4_ without O_2_ flow over Ni(5)/MOR-45 of 50 mg, and then estimated the amount of oxygen used during the reaction by the amount of the product, CO; the production of CO was found to be 16 μmol. The follows four reactions are the reactions involvement of oxygen in the oxidation of methane. Among them, if the production of CO is only based on Eq. (1), oxygen atom of 0.33 mmol/g is required to produce CO of 16 μmol. Besides, the amount of the remaining lattice oxygen in the catalyst after the reaction was calculated to be 0.36 mmol/g based on H_2_-TPR measurement. Hence, the total oxygen amount in the catalyst was estimated to be 0.69 mmol/g (=0.33 + 0.36 mmol/g). It should be noted that this value is almost accordance with that in the pristine catalyst (ca. 0.76 mmol/g), meaning that CH_4_ was oxidized to CO by using the lattice oxygen; only Eq. (1) proceeded with H_2_/CO ratio of 2.

1 $${\mathrm{CH}}_4 + \left[ {\mathrm{O}} \right] \to {\mathrm{CO}} + 2{\mathrm{H}}_2,$$

2 $${\mathrm{H}}_2 + \left[ {\mathrm{O}} \right] \to {\mathrm{H}}_2{\mathrm{O}},$$

3 $${\mathrm{CO}} + \left[ {\mathrm{O}} \right] \to {\mathrm{CO}}_2,$$

4 $${\mathrm{CH}}_4 + 4\left[ {\mathrm{O}} \right] \to {\mathrm{CO}}_2 + 2{\mathrm{H}}_2{\mathrm{O}}.$$

*[O]: lattice oxygen of NiO in the supported Ni catalyst.

**Fig. 7:**
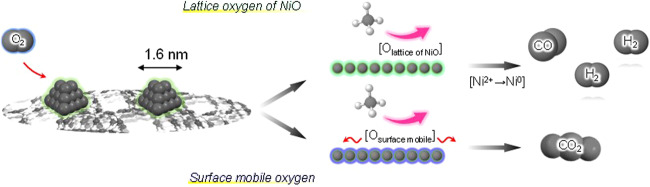
Schematic illustration of possible reaction mechanism based on the effect of surface oxygen species on the reaction. Oxidized CH_4_ is completely converted to CO and H_2_ with the H_2_/CO = 2.0 in the absence of surface mobile oxygen.

Based on the results, a possible reaction mechanism for the partial oxidation of methane to syngas production over Ni/MOR has been proposed. Before starting the reaction, Ni/MOR has highly dispersed NiO species with 1.6 nm in size. In the initial stage, CH_4_ was oxidized by using lattice oxygen atoms derived from NiO, and NiO was reduced to Ni (Ni^2+^ ⇄ Ni^0^). Oxidized CH_4_ was completely converted to CO and H_2_ with the H_2_/CO = 2.0 in the absence of surface mobile oxygen, i.e., reductive atmosphere (Fig. 7). Considering that this reaction did not proceed over Co/MOR^19^, the redox ability of the Ni species is one of the essential factors for selectively producing syngas from selective oxidation of methane. In addition, the successive oxidation of CO to CO_2_ could be greatly retarded by reducing the size of the NiO species, leading to the selective production of CO and H_2_. Thus proposed mechanism based on via redox cycle of NiO without the assistance of any surface mobile oxygen species is novel and the first report.

In conclusion, we succeeded in controlling the size and dispersion of Ni species by using zeolite as support. Thus zeolite–supported ultra-small sized Ni exhibited a high catalytic performance in the selective oxidation of CH_4_ to CO and H_2_, and it showed a high durability without formation of coke. From transient response experiment, we have found that methane was oxidized by the lattice oxygen of NiO in Ni/zeolite and CH_4_ oxidation proceeds under reductive atmosphere. We believe that our findings would give us important insights on the catalytic conversion of CH_4_.

## Methods

### Catalysts preparation

Zeolite supported Ni catalyst was prepared by a facile impregnation method based on incipient wetness technique. When the MOR-type zeolite was used as zeolite, the catalyst was designated as Ni(*x*)/MOR-*y*, where *x* and *y* were Ni loading amount and the Si/Al atomic ratio in zeolite, respectively. In a typical synthesis, the proton-type MOR-type zeolite with the Si/Al ratio of 45 (MOR-45, JRC-Z-HM90) was obtained from the Catalysis society of Japan(CSJ). Ni(NO_3_)_2_ (0.45 mmol) was dissolved in 10 mL of water. This solution was dropwisely added onto the MOR (0.5 g), and then the resulting wet solid was dried in air at 373 K, following calcination in air at 873 K for 6 h (rate of temperature increase, 10 K min^–1^).

### Catalysts characterization

Powder XRD patterns were recorded on a RINT-Ultima III (Rigaku) using a Cu Kα X-ray source (40 kV, 40 mA). XPS spectra were taken by using a ULVAC-PHI ESCA 1700R (Al Kα radiation). Binding energy was calibrated with respect to C 1 s peak of a carbon tape at 284.8 eV. Field-emission scanning electron microscopic images of the powder samples were obtained on a Hitachi SU9000 microscope operated at 1 kV. TEM images of the powder samples were obtained on a JEOL JEM-2010F. ^27^Al MAS NMR spectra were obtained on a JEOL ECA-600 spectrometer. Elemental analyses of the samples were performed on an inductively coupled plasma-atomic emission spectrometer (ICP-AES, Shimadzu ICPE-9000).

### Catalytic oxidation of methane

Catalytic oxidation of methane was performed in a continuous flow reactor under atmospheric pressure. After pretreatment of the catalyst (50 mg) at 873 K with Ar at the flow rate of 30 mL min^−1^ for 1 h, the mixture gas of CH_4_ (0.06 atm), O_2_ (0.03 atm), Ar (balance) at an SV 3.0 × 10^4^ mL h^−1^ g_-cat_^−1^ was fed into the reactor. The temperature inside the catalyst bed was monitored using thermocouple. The reaction products at the outlet of the reactor were analyzed by using on-line gas chromatography. CH_4_, CO, H_2_, and CO_2_ were analyzed by GC (Shimadzu, GC-2014) equipped with a thermal conductivity detector and packed column (Shimadzu, Shincarbon-ST 50/80, inner diameter 3 mm, length 6 m). For other products such as ethane and ethene, the other GC (Shimadzu, GC-2014) equipped flame ionization detector and a capillary column (Agilient, HP-PLOT Q, inner diameter 0.530 mm, length 30 m, film thickness 40.0 μm).

## Supplementary information

Supplementary Information

## Data Availability

The authors declare that all data supporting the findings of this study are available within the paper and its supplementary information files.

## References

[1] Hammond C, Conrad S, Hermances I (2012). Oxidative methane upgrading. ChemSusChem..

[2] Guo X (2014). Direct, nonoxidative conversion of methane to ethylene, aromatics, and hydrogen. Science.

[3] Sushkevich VL, Palagon D, Ranocchiari M, Bokhoven JAvan (2017). Selective anaerobic oxidation of methane enables direct synthesis of methanol. Science.

[4] Rofer-DePoorter CK (1981). A comprehensive mechanism for the Fischer-Tropsch synthesis. Chem. Rev..

[5] Li J (2018). Integrated tuneable synthesis of liquid fuels via Fischer–Tropsch technology. Nat. Catal..

[6] Böller B, Durner KM, Wintterlin J (2019). The active sites of a working Fischer–Tropsch catalyst revealed by operando scanning tunnelling microscopy. Nat. Catal..

[7] Wang P (2018). Synthesis of stable and low-CO_2_ selective ε-iron carbide Fischer-Tropsch catalysts. Sci. Adv..

[8] Van Hock JP (1980). Methane-steam reforming. Catal. Rev. Sci. Eng..

[9] Lulianeli A, Liguori S, Wilcox J, Basile A (2016). Advances on methane steam reforming to produce hydrogen through membrane reactors technology: A review. Catal. Rev. Sci. Eng..

[10] Abdulrasheed A (2019). A review on catalyst development for dry reforming of methane to syngas. Renew. Sustain. Energy Rev..

[11] Chein R-Y, Fung W-Y (2019). Syngas production via dry reforming of methane over CeO_2_ modified Ni/Al_2_O_3_ catalysts. Int. J. Hydrog. Energy.

[12] Enger BC, Lødeng R, Holmen A (2008). A review of catalytic partial oxidation of methane to synthesis gas with emphasis on reaction mechanisms over transition metal catalysts. Appl. Catal. A. Gen..

[13] Alvarez-Galvan C (2019). Partial oxidation of methane to syngas over nickel-based catalysts: influence of support type, addition of rhodium, and preparation method. Front. Chem..

[14] Ashcroft AT (1990). Selective oxidation of methane to synthesis gas using transition metal catalysts. Nature.

[15] Dissanayake D, Rosynek MP, Kharas KCC, Lunsford JH (1991). Partial oxidation of methane to carbon monoxide and hydrogen over a Ni/Al_2_O_3_ catalyst. J. Catal..

[16] Choudhary VR, Rajput AM, Prabhakar B (1993). Nonequilibrium oxidative conversion of methane to CO and H_2_ with high selectivity and productivity over Ni/Al_2_O_3_ at low temperatures. J. Catal..

[17] Torniainen PM, Chu X, Schmidt LD (1994). Comparison of monolith-supported metals for the direct oxidation of methane to syngas. J. Catal..

[18] Hou Y, Ogasawara S, Fukuoka A, Kobayashi H (2017). Zeolite-supported rhodium sub-nano cluster catalyst for low-temperature selective oxidation of methane to syngas. Catal. Sci. Technol..

[19] Hou Y, Nagamatsu S, Asakura K, Fukuoka A, Kobayashi H (2018). Trace mono-atomically dispersed rhodium on zeolite-supported cobalt catalyst for the efficient methane oxidation. Commun. Chem..

[20] Jin R (2000). Mechanism for catalytic partial oxidation of methane to syngas over a Ni/Al_2_O_3_ catalyst. Appl. Catal. A. Gen..

[21] Osuga R (2020). Metal cation-exchanged zeolites with the location, state, and size of metal species controlled. Chem. Commun..

[22] Takenaka S, Kobayashi S, Ogihara H, Otsuka K (2003). Ni/SiO_2_ catalyst effective for methane decomposition into hydrogen and carbon nanofiber. J. Catal..

[23] Vogt C, Kranenborg J, Monai M, Weckhuysen BM (2020). Structure sensitivity in steam and dry methane reforming over nickel: activity and carbon formation. ACS Catal..

[24] Barmparis GD, Lodziana Z, Lopez N, Remediakis IN (2015). Nanoparticle shapes by using Wulff constructions and first-principles calculations. Beilstein J. Nanotechnol..

[25] Richardson JT, Scates R, Twigg MV (2003). X-ray diffraction study of nickel oxide reduction by hydrogen. Appl. Catal. A. Gen..

[26] Nørskov JK (2004). Origin of the overpotential for oxygen reduction at a fuel-cell cathode. J. Phys. Chem. B.

