# Supplementary material for: Zeolite-supported ultra-small nickel as catalyst for selective oxidation of methane to syngas
Source: Commun Chem. 2020 Sep 16;3:129. doi: 10.1038/s42004-020-00375-0 (PMC9814408; doi:10.1038/s42004-020-00375-0)
Supplement: Supplementary file 1 — Supplementary Information [file 42004_2020_375_MOESM1_ESM.pdf]

Zeolite-supported ultra-small nickel as catalyst for selective oxidation of methane to syngas

Shuhei Yasuda[1], Ryota Osuga[1], Yusuke Kunitake[1], Kazuya Kato[2], Atsushi Fukuoka[2], Hirokazu Kobayashi[2], Min Gao[2], Jun-ya Hasegawa[2], Ryo Manabe[3], Hisashi Shima[3], Susumu Tsutsuminai[3], and Toshiyuki Yokoi[1]*

[1] *Institute for Innovative Research, Tokyo Institute of Technology, 4259 Nagatsuta, Midori-ku, Yokohama 226-8503, Japan.*

[2] *Institute for Catalysis, Hokkaido University, kita 21 Nishi 10, Kita-ku, Sapporo,001-0021, Japan.*

[3] *Mitsubishi Chemical Corporation, 1000 Kamoshida-cho, Aoba-ku, Yokohama 227-8502, Japan**

corresponding author: yokoi@cat.res.titech.ac.jp

S1. Supplementary Methods

S1.1. Reagents

All reagents were used without further purification. $Ni(NO_3)_2 \cdot 6H_2O$ was purchased from Wako.

S1.2. Catalysts preparation

Zeolite supported Ni catalyst

The influences of Al content in the zeolite on the state of Ni species loaded and the catalytic performance were investigated. The catalysts with the Si/Al ratio varied were prepared with the Ni loading amount kept (x = 5); the catalyst was designated as "Ni(5)/MOR-y, where y was the Si/Al ratio between 7.5 and 120. Ni(5)/MOR-7.5 (MOR-7.5, JRC-Z-HM15, Si/Al = 7.5), Ni(5)/MOR-10 (MOR-10, JRC-Z-HM20, Si/Al = 10), or Ni(5)/MOR-120 (MOR-120, HSZ-690HOA, Si/Al = 120, Tosoh), were prepared by using the incipient wetness impregnation procedure but instead of using MOR-45.

As a screening of zeolite structure, the MFI- or FER-type aluminosilicate was used instead of MOR. The proton-type MFI with Si to Al mol ratio of 45 (MFI-45, JRC-Z-90H) was obtained from

CSJ. The ammonium-type FER with Si to Al mol ratio of 10 (FER-10, CP914C) was purchased from ZEOLYST international. The powder was calcined at 873 K for 6 h. Ni/MFI-45 or Ni/FER-10 was prepared by using incipient wetness impregnation procedure but instead of using MOR-45.

Ni/SiO_2-Al_2O_3, Ni/SiO_2

As a control, amorphous silica-alumina-supported or amorphous silica-supported Ni (5 wt%, Ni/SiO_2) was also prepared and evaluated to clarify the importance of the use of zeolite as support. The amorphous SiO_2 (M7D) was purchased from CABOT. The amorphous SiO_2-Al_2O_3 (JRC-SAH-1) was obtained from CSJ. Ni/SiO_2-Al_2O_3, Ni/SiO_2 was prepared by using incipient wetness impregnation procedure but instead of using MOR-45.

Supported Ni catalysts were prepared by using evaporated–impregnation procedure

As a control, supported Ni catalysts were prepared by an impregnation method and evaluated to clarify the importance of the impregnation method and Ni particle size. $Ni(NO_3)_2$ was dissolved in water, and then support was added into the solution and the suspension was stirred at room temperature for 24 h. The resulting suspension was evaporated and dried 383 K overnight following by calcination at 823 K in air for 6 h. Ni(x)/MOR-45(x =0.1–10), Ni(3)/SiO_2, Ni(3)/Al_2O_3, or Ni(3)/CeO_2 was prepared by using impregnation procedure but instead of using incipient wetness impregnation procedure in **S1.2**.

S1.3. Catalytic oxidation of methane

The carbon balance in the reaction was calculated based on Supplementary Equation 1 (S. Eq. 1) and was 100 ± 1% for whole the reaction.

$$\text{Carbon balance (\%)} = \frac{\text{Moles of total carbon in products}}{\text{Moles of carbon in substrate}} \times 100 \qquad \text{(S. Eq. 1)}$$

The yield of and selectivity to each product were calculated based on carbon basis with the following Supplementary equations (S. Eq. 2)-(S. Eq. 5).

$$\text{Conversion of substrate (\%)}=\frac{\text{Moles of substrate consumed}}{\text{Moles of substrate fed}}\times 100 \qquad \text{(S. Eq. 2)}$$

$$\text{Yield of a product (\%)}=\frac{\text{Moles of total carbon in a product defined}}{\text{Moles of carbon in substrate}} \qquad \text{(S. Eq. 3)}$$

$$\text{Selectivity to a product (\%)}=\frac{\text{Moles of total carbon in product defined}}{\text{Moles of carbon in substrate consumed}}\times 100 \qquad \text{(S. Eq. 4)}$$

$$H_2/CO=\frac{\text{Moles of } H_2}{\text{Moles of CO}}\times 100 \qquad \text{(S. Eq. 5)}$$

To observe transient response of the catalytic performance, the feed gas was promptly switched from to another, while the reaction temperature and the SV were kept constant.

Catalytic performance was evaluated under the following conditions; CH_4:O_2:Ar = 0.50:0.04:0.46 (atm); temperature, 873 K; catalyst, 50 mg; SV, 6.0×10^4 mL h^{-1} g_{-cat}^{-1}; and total pressure = 0.1 MPa, but instead of reaction condition introduced before.

S1.4. Computational Details

The calculations were carried out using density functional theory (DFT) in the generalized-gradient approximation (GGA) with the functional of Perdew, Burke and Ernzerh (PBE)[1] as implemented in the Vienna *ab initio* Simulation Package code (VASP 5.41).[2-3] The Vander-Waals interactions were considered by using DFT-D3 method[4] implemented in VASP. The $3d^9 4s^1$, $2s^2 2p^4$, $2s^2 2p^2$, and $1s^1$ of Ni, O, C and H atoms were treated for valence electrons, respectively. To reproduce the magnetic properties of NiO, the Hubbard U correction with a value of 5.3 eV was applied for *d* electrons of Ni atoms. The calculated lattice parameters of bulk NiO are a = b = c = 4.169 Å, and a magnetic moment on the Ni ions of M = 1.69 μ_B which is consistent with the experimental value a = b = c = 4.177 Å,[5] and M = 1.90 μ_B.[6] The NiO(100) surface with 4×4×4 element of unit cell was chosen as the slab model and 15 Å of vacuum was used to separate the slabs. The bottom two layers were fixed to represent the bulk and the other layers were fully relaxed during optimization. The periodic boundary conditions were used for all systems. All calculations were spin polarized. The

NiO cluster was represented by 2×2×2 element unit cell. Various directions of magnetic moments were tested. The most stable state contains Ni atoms with opposite magnetic moment direction at the transposition of an oxygen atom. The total magnetic moments are 0.0 μ_B for NiO(100) surface and 4.0 μ_B for NiO cluster. The energy cutoff of 450 eV was chosen to guarantee convergence of the total energies and forces. The SCF tolerance was set to 1.0 e^{-4} per atoms, and the Gaussian smearing scheme with a width of $\sigma = 0.05$ eV was used. The force tolerance was set to 0.03 eV/Å for geometry optimization. All the calculations were performed at Γ point of the Brillouin zone. The surface energy is calculated by the following equation (S. Eq. 6),

$$E_{\text{surface}} = \frac{E_{\text{slab}} - N * E_{\text{bulk}}}{2A} \quad \text{(S. Eq. 6)}$$

where E_{slab} is the electronic energy of the calculated slab model, E_{bulk} is total energy of the bulk NiO unit cell, N is the coefficient to normalize the bulk unit cell energy to the same number of atoms as the slab model, and A is the slab surface area.

Supplementary References

1. Perdew, J. P., Burke, K., Ernzerhof, M., Generalized Gradient Approximation Made Simple. *Phys. Rev. Lett.* **77**, 3865 (1996).
2. Kresse, G., Furthmüller, J., Efficiency of ab-initio total energy calculations for metals and semiconductors using a plane-wave basis set. *Comput. Mater. Sci.* **6**, 15−50 (1996).
3. Kresse, G., Hafner, J., Ab initio molecular dynamics for liquid metals. *Phys. Rev. B* **47**, 558(R) (1993).
4. Grimme, S., Antony J., Ehrlich, S., Krieg, H., A consistent and accurate ab initio parametrization of density functional dispersion correction (DFT-D) for the 94 elements H-Pu. *J. Chem. Phys.* **132**, 154104 (2010).
5. Fiévet, F., Germi, P., de Bergevin, F., Figlarz, M. J., Lattice parameter, microstrains and non-stoichiometry in NiO. Comparison between mosaic microcrystals and quasi-perfect single microcrystals. *J. Appl. Cryst.* **12**, 387−394 (1979).
6. Cheetham, A. K., Hope, D. A. O., Magnetic ordering and exchange effects in the antiferromagnetic solid solutions **$Mn_xNi_{1-x}O$.** *Phys. Rev. B* 27, 6964 (1983).

Supplementary Table 1 Catalytic data for oxidative conversion of CH_4 over various catalysts prepared by simple impregnation procedure.[a]

Entry	Catalyst	Conversion (%)	Seletivity (%)		Yield (C-mol%)		Yield (H-mol%)	Ni Particle size from XRD (nm)[b]
		CH_4	CO	CO_2	CO	CO_2	H_2	
1	Ni(0.5)/MOR–45-evap	2.6	34	66	0.88	1.7	0.41	-
2	Ni(1)/MOR–45-evap	3.9	17	83	0.68	3.3	0.58	-
3	Ni(3)/MOR–45-evap	9.4	79	21	7.4	2.0	13	3[c]
4	Ni(5)/MOR–45-evap	30	27	4.0	8.0	1.2	28	17
5	Ni(3)/SiO_2-evap	1.4	0	100	0	1.4	0	17
6	Ni(3)/Al_2O_3-evap	3.4	74	26	2.5	0.89	0.87	-
7	Ni(3)/CeO_2-evap	1.3	0	100	0	1.3	0	23

[a] Reaction conditions: CH_4:O_2:Ar = 0.50:0.04:0.46 (atm); temperature, 873 K; W/F, 0.75 g_{-cat} h mol^{-1}; SV, 6.0×10^4 mL h^{-1} g_{-cat}^{-1}; and total pressure = 0.1 MPa.

[b] Ni particle(NiO(111)) size was calculated by Scherrer's equation.

[c] Ni particle size was estimated from HAADF STEM

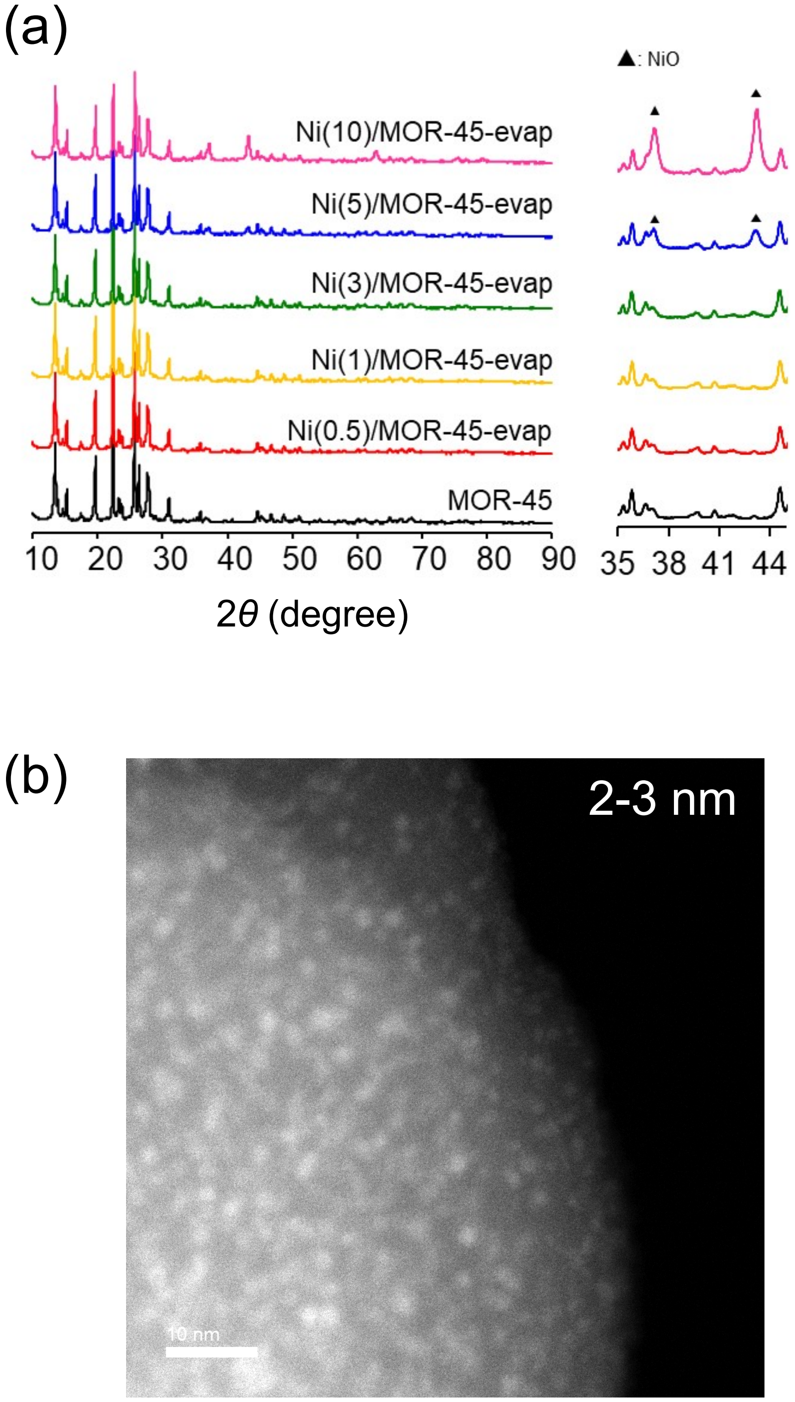

Supplementary Figure 1 (a) XRD patterns of Ni(*x*)/MOR-45-evap, which were prepared by the impregnation method based on an evaporation technique. (b) High-angle annular dark-field scanning transmission electron microscopy image of Ni(3)/MOR-45-evap. Scale bar: 10 nm.

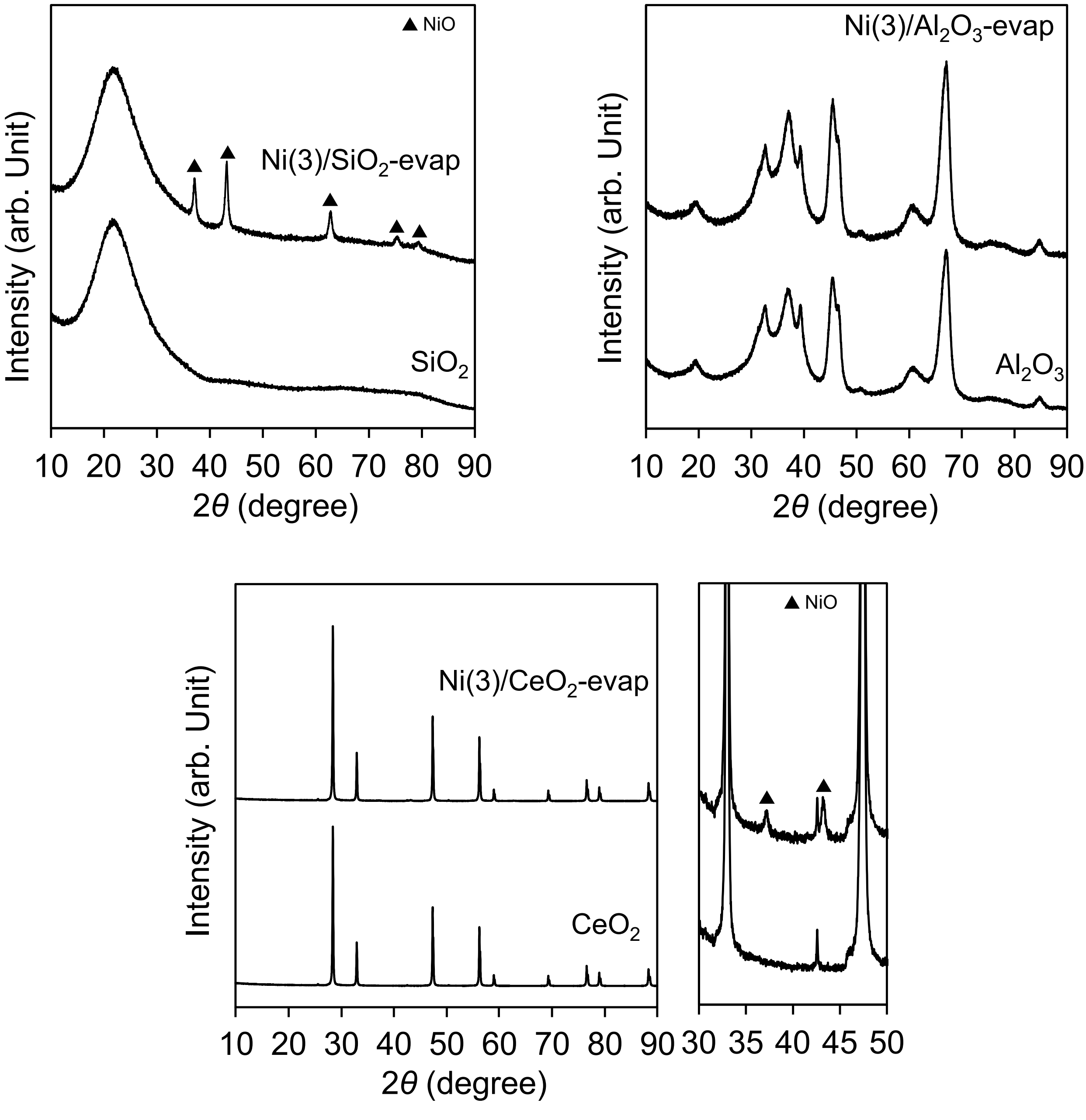

Supplementary Figure 2 XRD patterns of supported Ni catalysts which were prepared by evaporation–impregnation procedure.

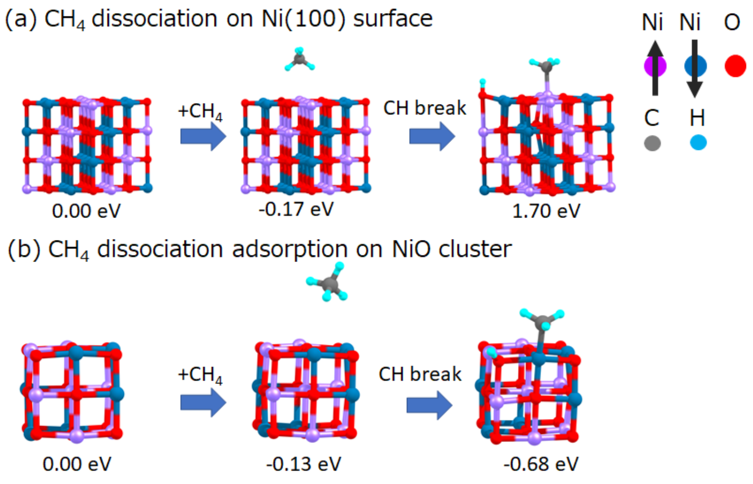

Supplementary Figure 3 The dissociative adsorption of CH_4 on NiO(100) surface (a) and NiO cluster (b). The purple and blue cycle indicted the Ni atoms with up and down spins, respectively. The energy is calculated by equation, $E_b = E_{tot} - E_{cat} - E_{CH_4}$, where E_{tot}, E_{cat}, and E_{CH_4} indicate the total energy of total system, NiO surface/cluster, and CH_4, respectively.

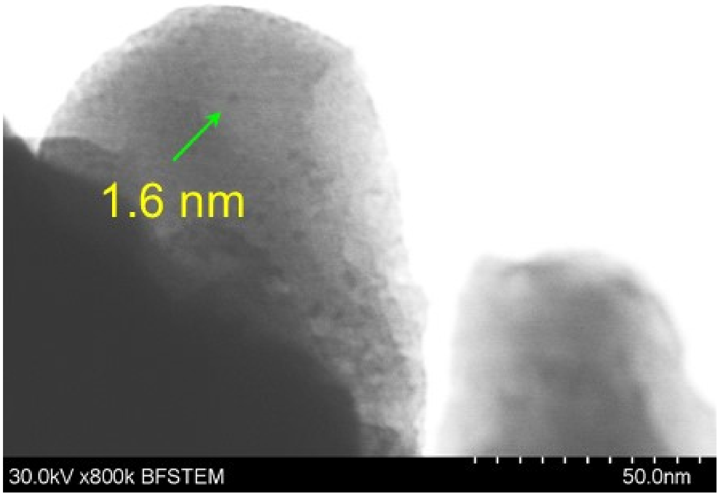

Supplementary Figure 4 The STEM image of Ni(5)/MOR-45.

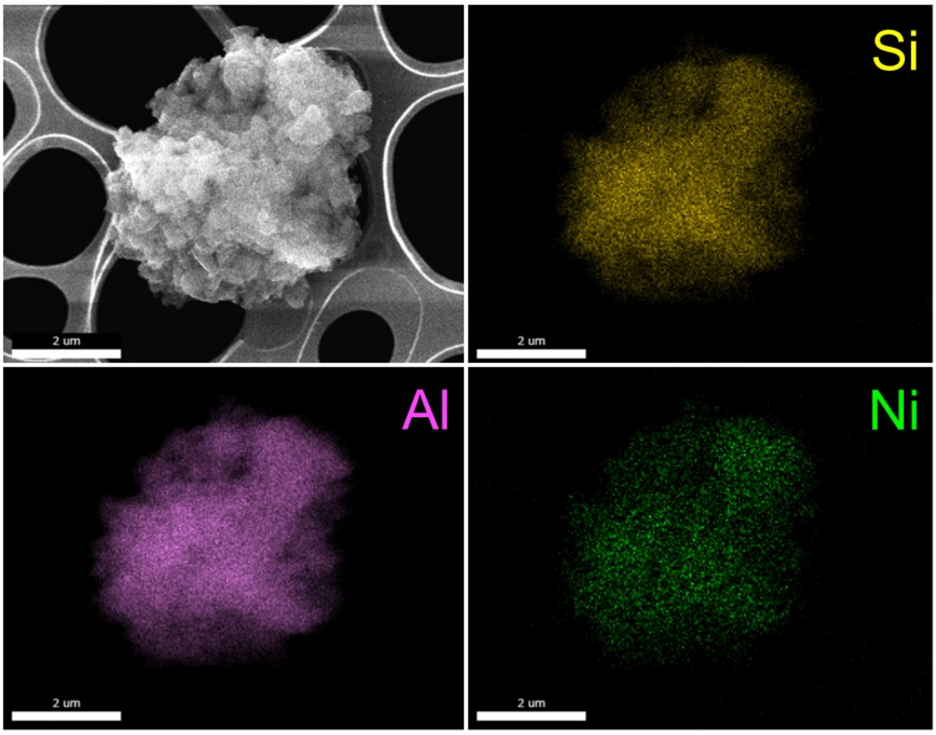

Supplementary Figure 5 SEM image and EDX mappings of Ni(5)/MOR-45.

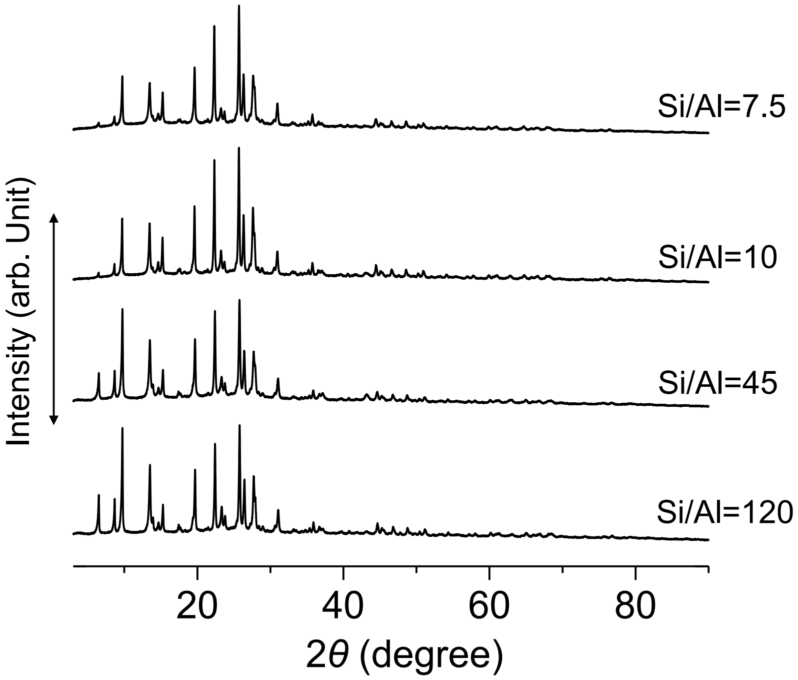

Supplementary Figure 6 XRD patterns of Ni(5)/MOR-*y*.

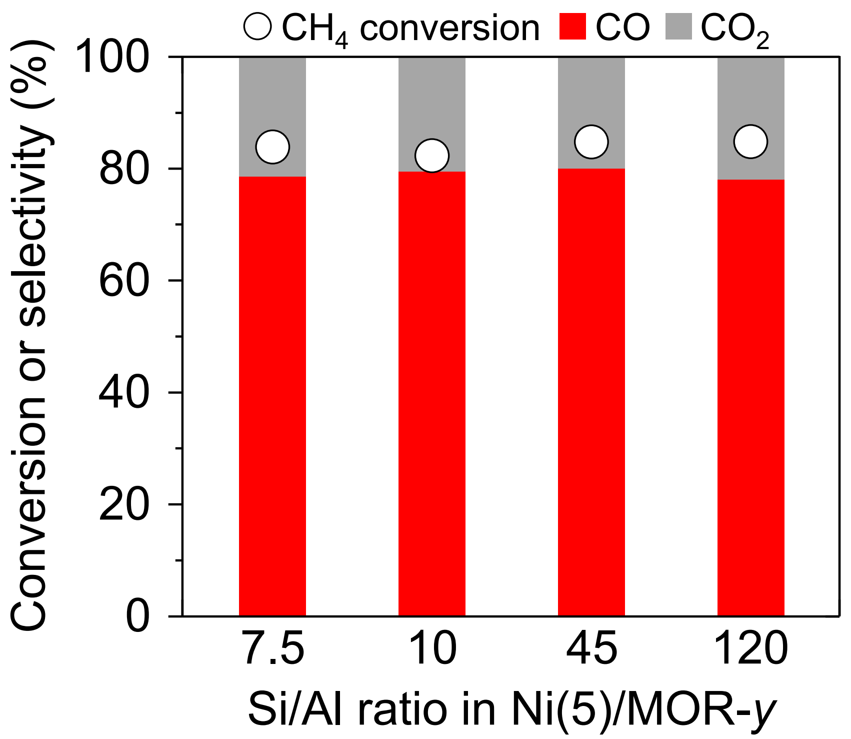

Supplementary Figure 7 Oxidation of methane over Ni(5)/MOR-*y*, where *y* is the Si/Al ratio, 7.5 10, 45, 120. (○) Conversion of methane, (red and gray) bars represent the yields of CO and CO_2, respectively. Reaction conditions: CH_4:O_2:Ar = 0.06:0.03:0.91 (atm); total pressure, 0.1 MPa; temperature, 873 K; and SV = 3.0×10^4 mL h^{-1} g_{-cat}^{-1}. Catalytic data were taken after at least 3 h from the beginning of the reaction.

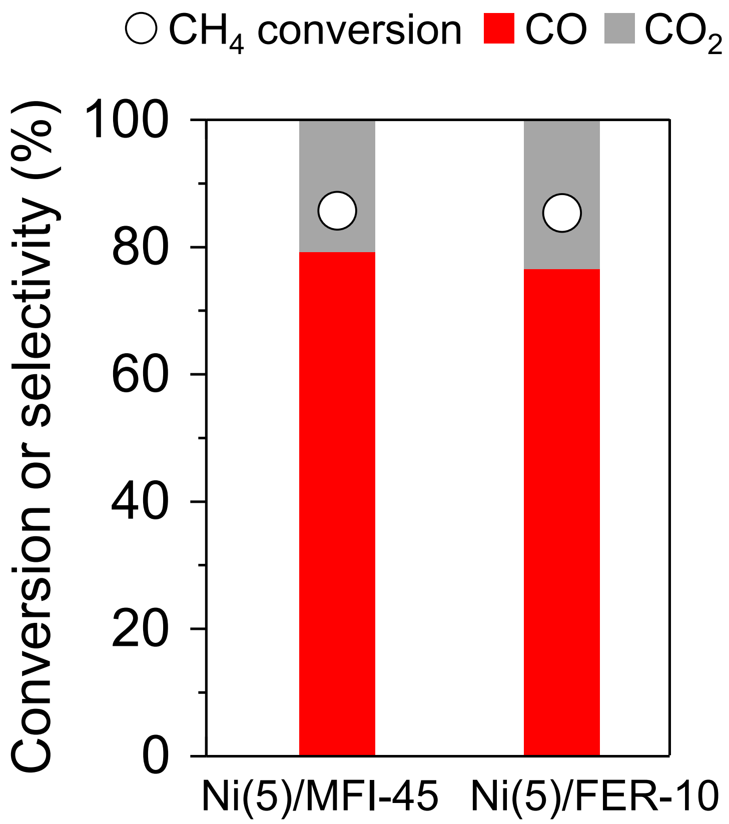

Supplementary Figure 8 The oxidation of methane over Ni(5)/MFI-45 or Ni(5)/FER-10. (○) Conversion of methane, (red and gray) bars represent selectivities to CO and CO_2, respectively. Reaction conditions: CH_4:O_2:Ar = 0.06:0.03:0.91 (atm); total pressure, 0.1 MPa; temperature, 873 K; and SV = 3.0×10^4 mL h^{-1} g_{-cat}^{-1}. Catalytic data were taken after at least 3 h from the beginning of the reaction.

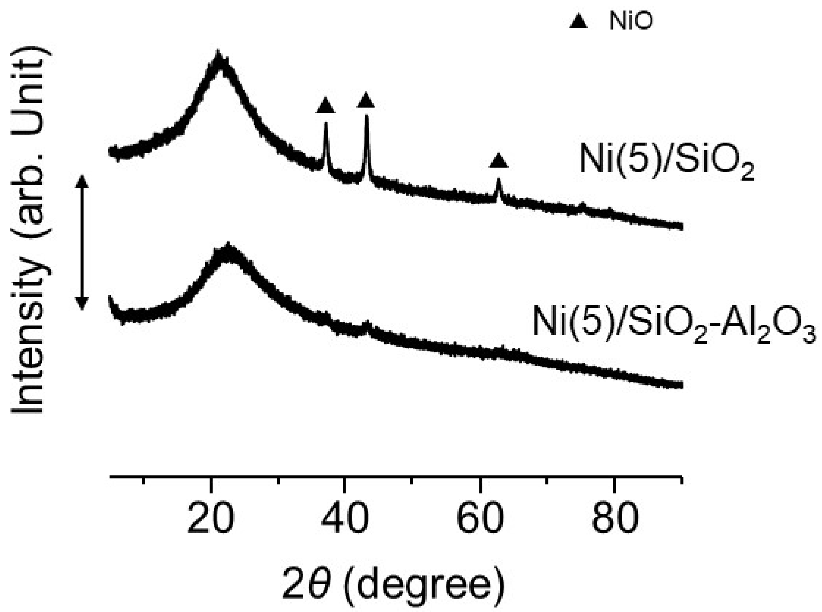

Supplementary Figure 9 XRD patterns of supported Ni catalysts, Ni(5)/SiO_2 and Ni(5)/SO_2-Al_2O_3.

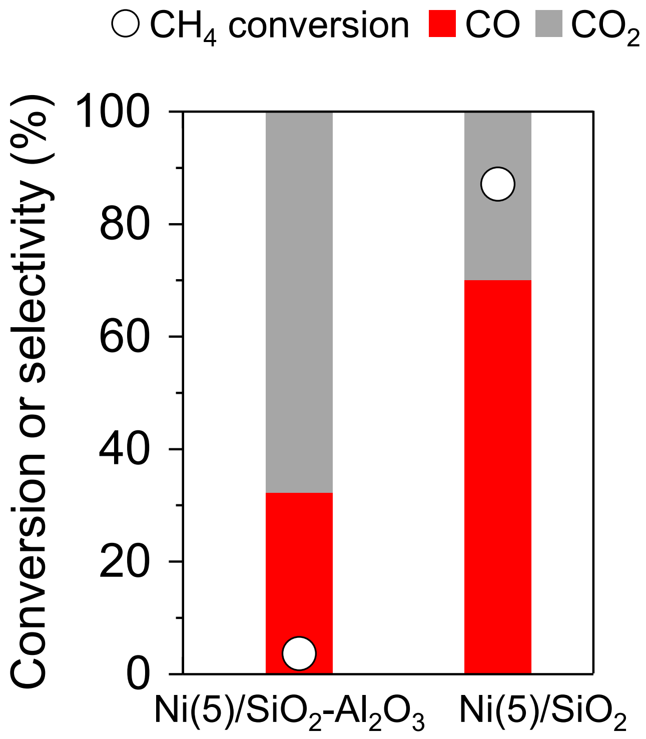

Supplementary Figure 10 The oxidation of methane over Ni(5)/SiO_2-Al_2O_3 or Ni(5)/SiO_2. (○) Conversion of methane, (red and gray) bars represent selectivities to CO and CO_2, respectively. Reaction conditions: CH_4:O_2:Ar = 0.06:0.03:0.91 (atm); total pressure, 0.1 MPa; temperature, 873 K; and SV = 3.0 × 10^4 mL h^{-1} g_{-cat}^{-1}. Catalytic data were taken after at least 3 h from the beginning of the reaction.

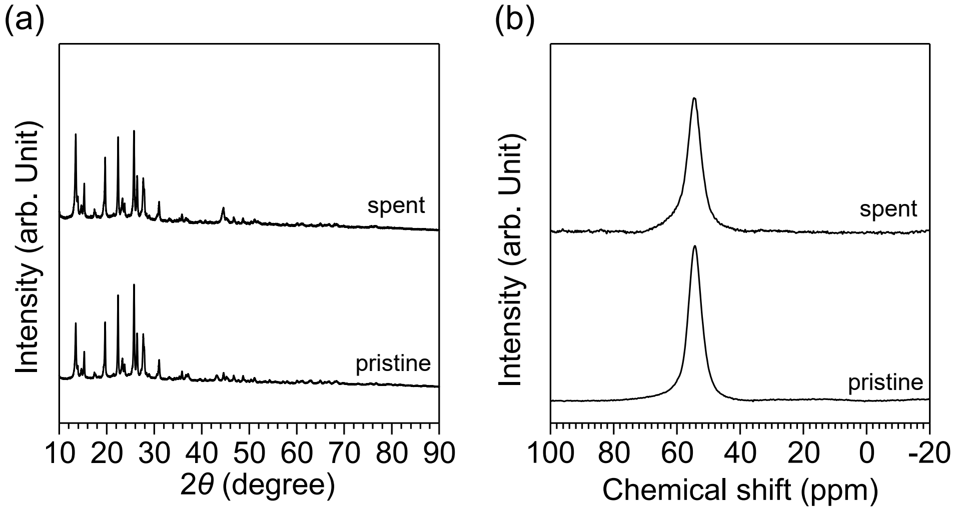

Supplementary Figure 11 XRD patterns and ^{27}Al MAS NMR spectra of the Ni(5)/MOR-45 and Ni(5)/MOR-45 sample after the reaction at 873 K(Figure 1) Reaction conditions: CH_4:O_2:Ar = 0.06:0.03:0.91 (atm); total pressure, 0.1 MPa; temperature, 873 K; and SV = 3.0 × 10^4 mL h^{-1} g_{-cat}^{-1}.

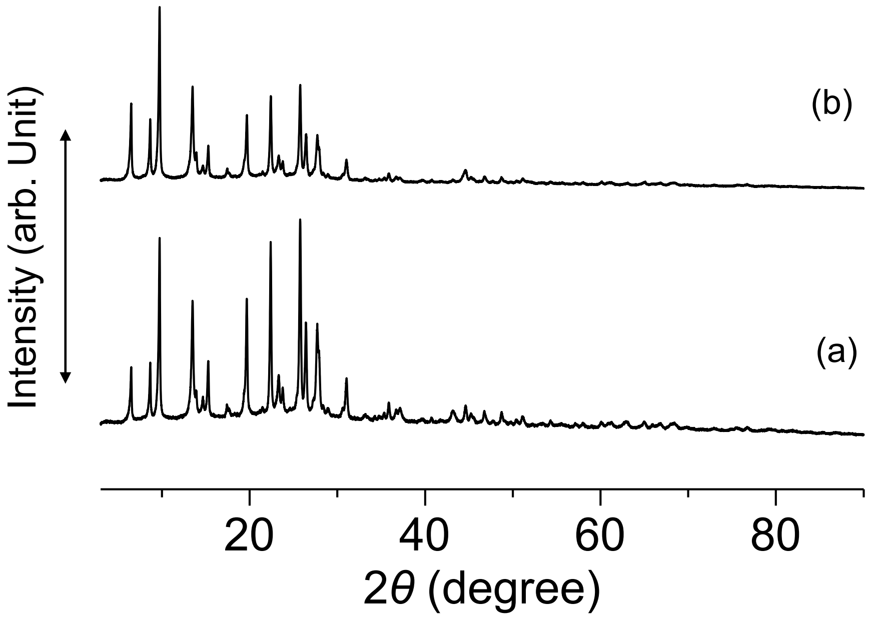

Supplementary Figure 12 XRD patterns of (a) pristine Ni(5)/MOR-45 and (b) the Ni(5)/MOR-45 sample after the reaction at 973 K for 8000 min (Figure 4). Reaction conditions: CH_4:O_2:Ar =0.06:0.03:0.91 (atm); total pressure, 0.1 MPa; temperature, 973 K; and SV = 3.0 × 10^4 mL h^{-1} g_{-cat}^{-1}.

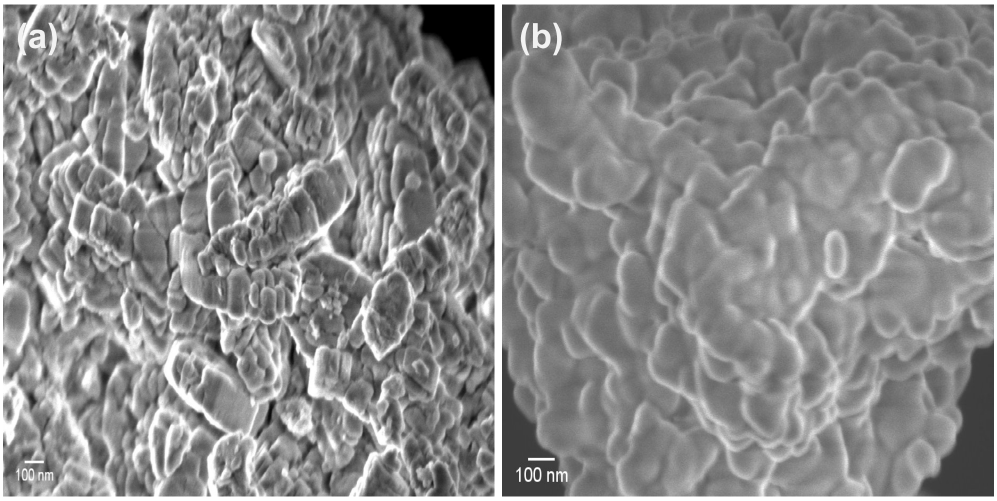

Supplementary Figure 13 SEM images of (a) pristine Ni(5)/MOR-45 and (b) the Ni(5)/MOR-45 sample after the reaction at 973 K for 8000 min (Figure 4). Reaction conditions: CH_4:O_2:Ar = 0.06:0.03:0.91 (atm); total pressure, 0.1 MPa; temperature, 973 K; and SV = 3.0 × 10^4 mL h^{-1} g_{-cat}^{-1}. Scale bar: 100 nm.

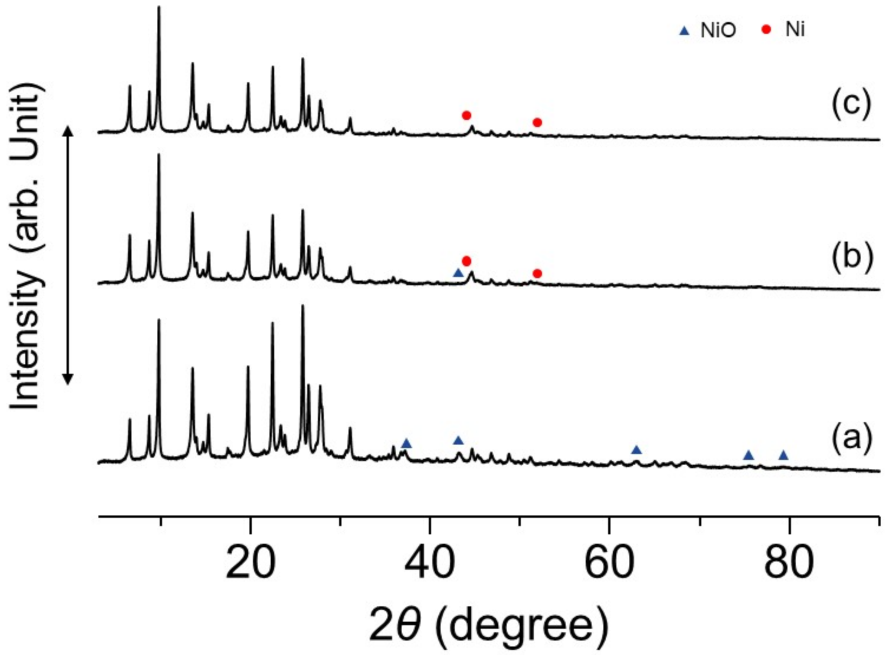

Supplementary Figure 14 XRD patterns of the Ni(5)/MOR-45 catalyst experienced in the transient responses (Figure 5) at 873 K. (a) pristine, (b) the catalyst obtained for the reaction in the presence of oxygen and (c) that of in the absence of oxygen.

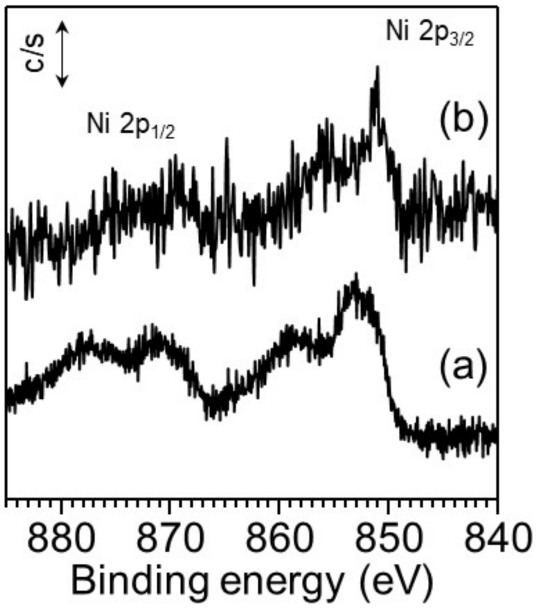

Supplementary Figure 15 X-ray photoelectron spectra recorded with Ni(5)/MOR-45 experienced in the transient responses in Figure 5 at 873 K. (a) pristine, the sample of (b) was obtained for the reaction in the absence of oxygen.

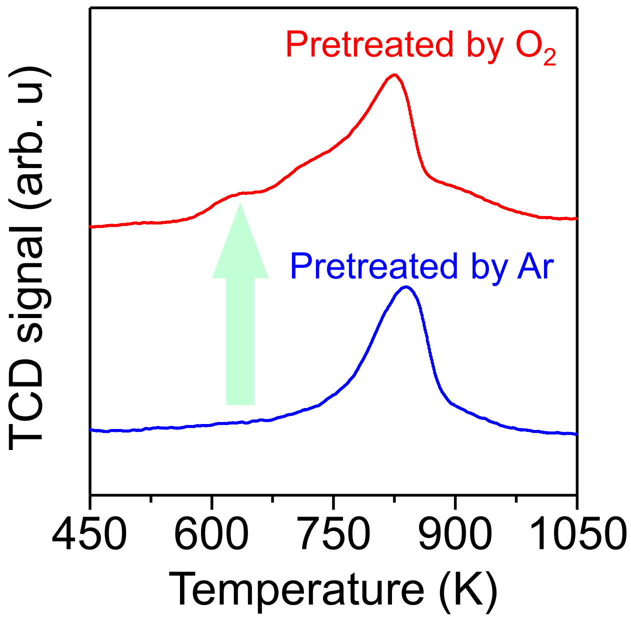

Supplementary Figure 16 TPR profiles of Ni(5)/MOR-45.

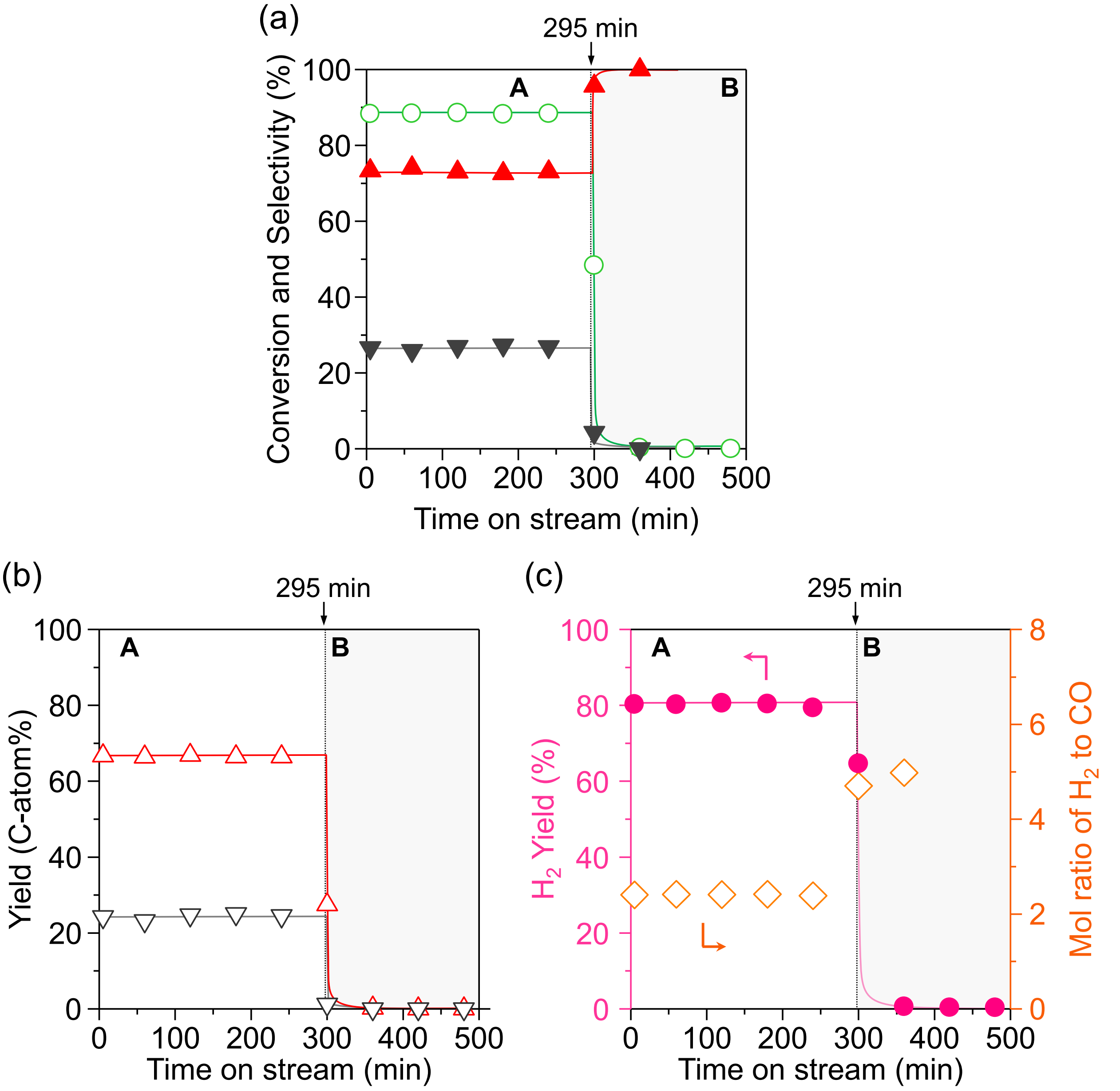

Supplementary Figure 17 Transient response for the oxidation of methane over Ni(5)/SiO_2 by changing the feed gas from (A) in the presence of O_2 (P_{O2} = 0.03 atm) to (B) in the absence of O_2 (P_{O2} = 0 atm) at 295 min. (**a**) Conversion of (○) methane and selectivities for (▲) CO and (▼) CO_2. (b) Yields of (△) CO and (▽) CO_2. (**c**) (●) Yield of H_2 and (◊) mol ratio of H_2 to CO. Reaction conditions: CH_4:O_2:Ar = (A) 0.06:0.03:0.91 (atm), (B) 0.06:0:0.94 (atm); total pressure, 0.1 MPa; temperature, 873 K; and SV = 3.0 × 10^4 mL h^{-1} g_{-cat}^{-1}.

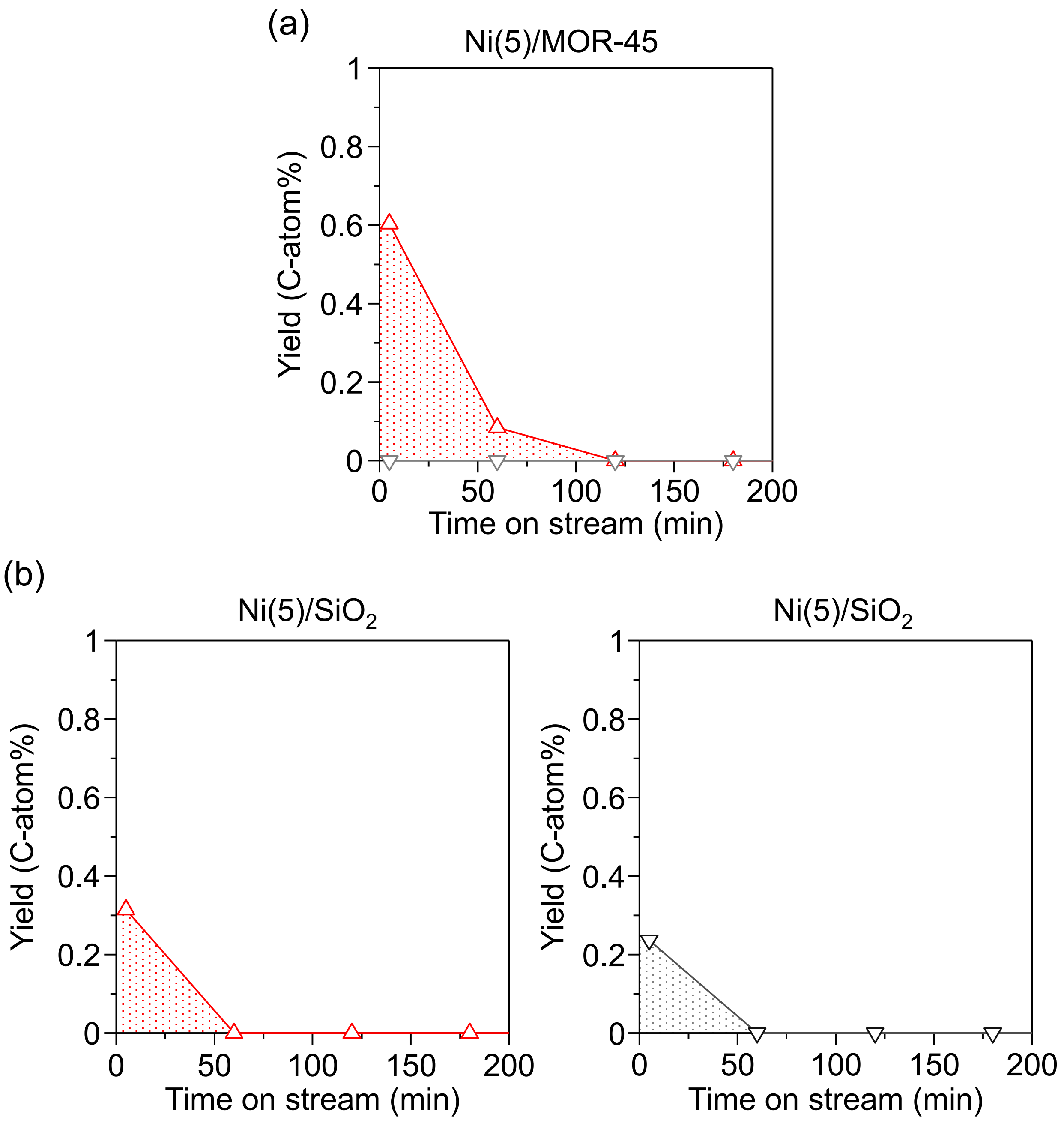

Supplementary Figure 18 The change in yield of the products along with time on stream. The area is related to the consumption of lattice oxygen originated from the NiO species in (a) Ni(5)/MOR-45 and (b) Ni(5)/SiO_2. Yields of (△) CO and (▽) CO_2. Reaction conditions: CH_4:O_2:Ar = 0.06:0:0.94 (atm); total pressure, 0.1 MPa; temperature, 873 K; and SV = 3.0×10^4 mL h^{-1} g_{-cat}^{-1}.
